# Supplementary material for: Impact of Nystatin Oral Rinse on Salivary and Supragingival Microbial Community among Adults with Oral Candidiasis
Source: Microorganisms. 2023 Jun 5;11(6):1497. doi: 10.3390/microorganisms11061497 (PMC10303349; doi:10.3390/microorganisms11061497)
Supplement: Supplementary file 1 [file microorganisms-11-01497-s001.zip › microorganisms-2402372-supplementary.pdf]

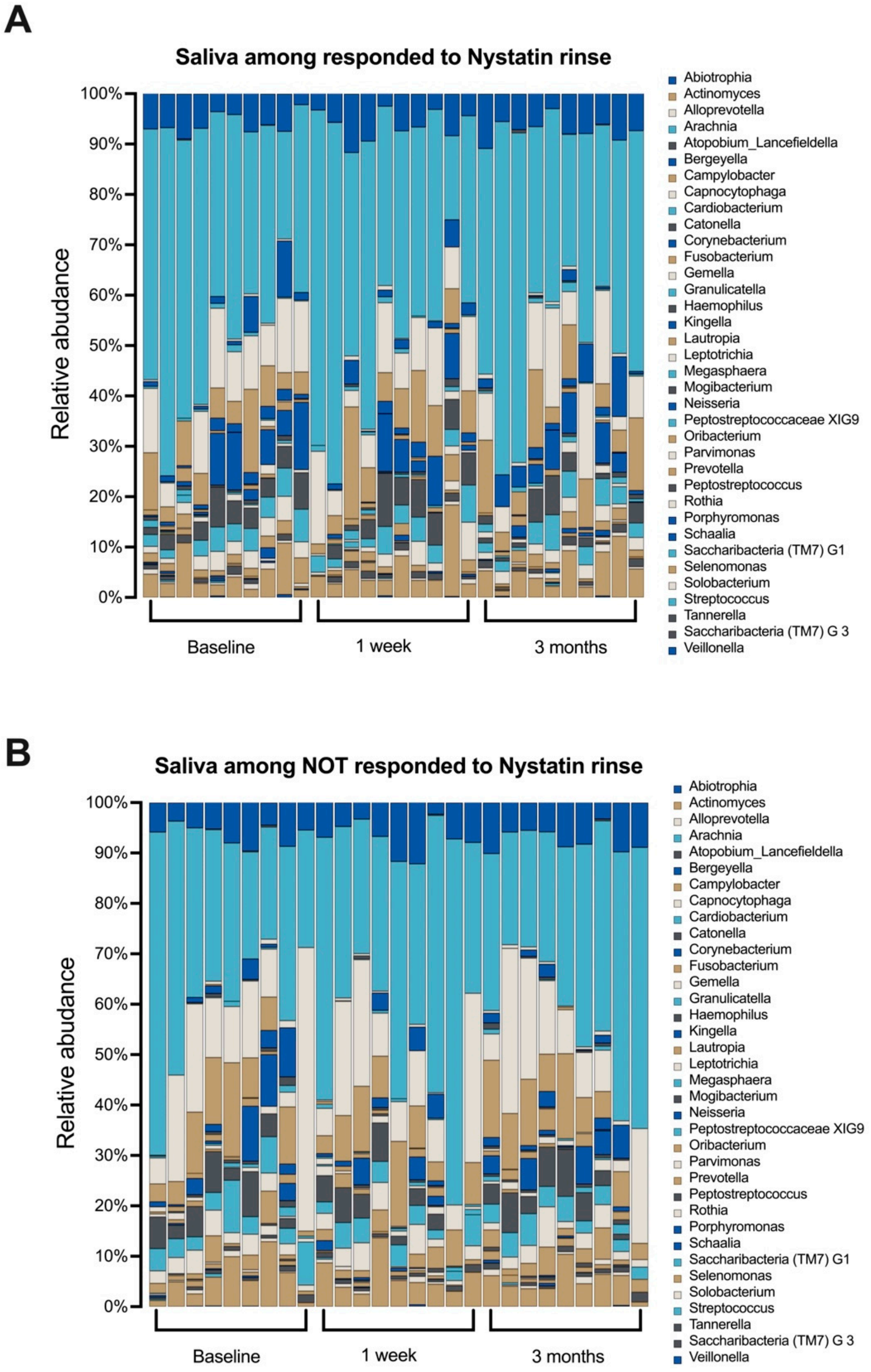

Figure S1. Relative Abundance of Genera in Saliva Samples Relative abundance of genera in saliva samples from the subjects who responded to Nystatin rinse (A) and who did not (B). The bars are organized based on the visit (baseline, 1 week, and 3 months). Top 38 most abundant genera were shown in the figures.

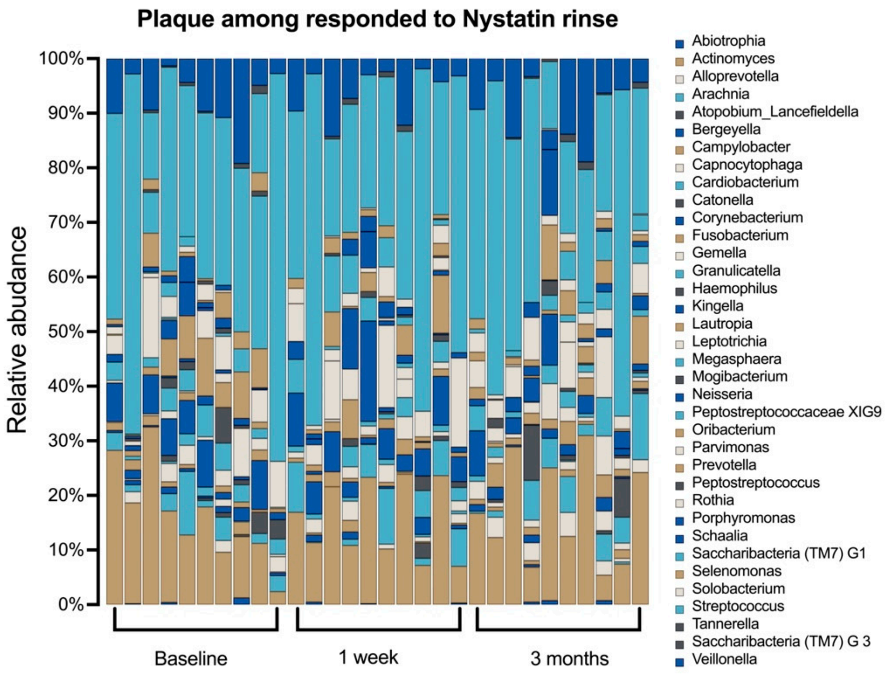

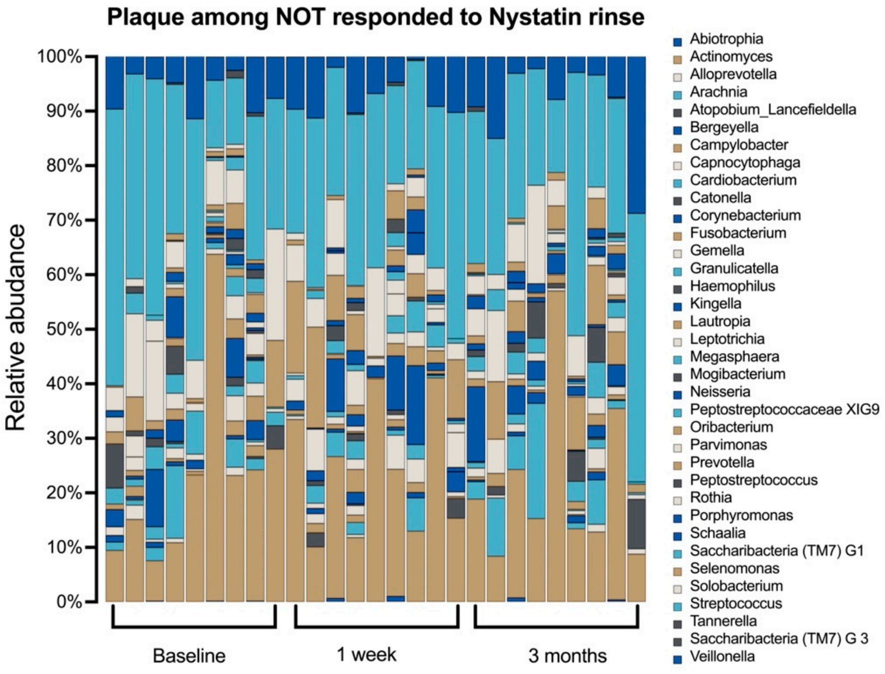

Figure S2. Relative Abundance of Genera in Saliva Samples. Relative abundance of genera in plaque samples from the individuals who responded to Nystatin rinse (A) and who did not (B). The bars are organized based on the visit (baseline, 1 week, and 3 months). Top 38 most abundant genera were shown in the figures.

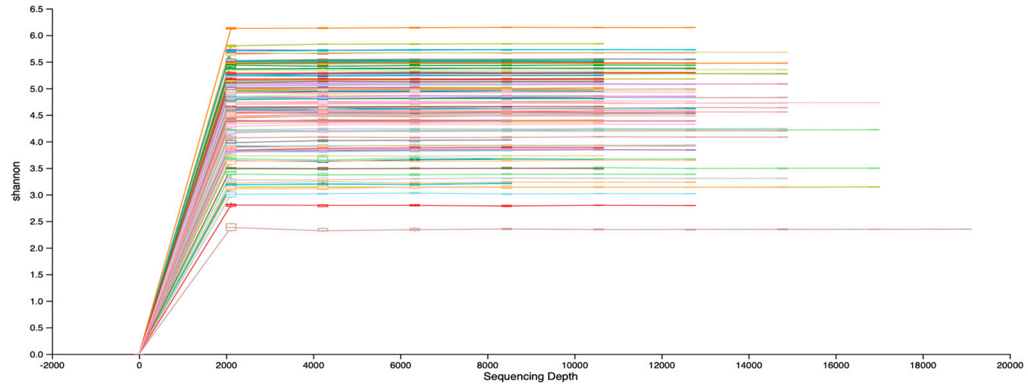

Figure S3. Rarefare curve of saliva and dental plaque samples. Diversity measures are affected by the sampling depth. Rarefaction calculated the species richness for a given number of individual samples, based on the construction of rarefaction curve. This rarefaction curve plotted the number of species as a function of the number of samples. species are found, but the curves plateau as only the rarest species remain to be sampled.
